# Cardiovascular outcome associations among cardiovascular magnetic resonance measures of arterial stiffness: the Dallas heart study

**DOI:** 10.1186/1532-429X-16-33

**Published:** 2014-05-14

**Authors:** Christopher D Maroules, Amit Khera, Colby Ayers, Akshay Goel, Ronald M Peshock, Suhny Abbara, Kevin S King

**Affiliations:** 1 Departments of Radiology, University of Texas Southwestern Medical Center, 5323 Harry Hines Boulevard, Dallas, TX 75390-8896, USA; 2 Division of Cardiology, Internal Medicine, and Clinical Sciences, Dallas, TX, USA; 3 Donald W. Reynolds Cardiovascular Clinical Research Center, University of Texas Southwestern Medical Center, Dallas, TX, USA

**Keywords:** Arterial stiffness, Pulse wave velocity, Aortic distensibility, Total arterial compliance, Cardiovascular magnetic resonance

## Abstract

**Background:**

Cardiovascular magnetic resonance (CMR) has been validated for the noninvasive assessment of total arterial compliance and aortic stiffness, but their associations with cardiovascular outcomes is unknown. The purpose of this study was to evaluate associations of CMR measures of total arterial compliance and two CMR measures of aortic stiffness with respect to future cardiovascular events.

**Methods:**

The study consisted of 2122 Dallas Heart Study participants without cardiovascular disease who underwent CMR at 1.5 Tesla. Aortic stiffness was measured by CMR-derived ascending aortic distensibility and aortic arch pulse wave velocity. Total arterial compliance was calculated by dividing left ventricular stroke volume by pulse pressure. Participants were monitored for cardiovascular death, non-fatal cardiac events, and non-fatal extra-cardiac vascular events over 7.8 ± 1.5 years. Cox proportional hazards regression was used to assess for associations between CMR measures and cardiovascular events.

**Results:**

Age, systolic blood pressure, and resting heart rate were independently associated with changes in ascending aortic distensibility, arch pulse wave velocity, and total arterial compliance (all p < .0001). A total of 153 participants (6.9%) experienced a cardiovascular event. After adjusting for traditional risk factors, total arterial compliance was modestly associated with increased risk for composite events (HR 1.07 per 1SD, p = 0.03) while the association between ascending aortic distensibility and composite events trended towards significance (HR 1.18 per 1SD, p = 0.08). Total arterial compliance and aortic distensibility were independently associated with nonfatal cardiac events (HR 1.11 per 1SD, p = 0.001 and HR 1.45 per 1SD, p = 0.0005, respectively), but not with cardiovascular death or nonfatal extra-cardiac vascular events. Arch pulse wave velocity was independently associated with nonfatal extra-cardiac vascular events (HR 1.18 per 1SD, p = 0.04) but not with cardiovascular death or nonfatal cardiac events.

**Conclusions:**

In a multiethnic population free of cardiovascular disease, CMR measures of arterial stiffness are associated with future cardiovascular events. Total arterial compliance and aortic distensibility may be stronger predictors of nonfatal cardiac events, while pulse wave velocity may be a stronger predictor of nonfatal extra-cardiac vascular events.

## Background

Arterial stiffening is one of the earliest detectable pathologic changes within the artery wall and is a strong predictor of future cardiovascular events and mortality [1,2]. Carotid-femoral pulse wave velocity by applanation tonometry is the most widely reported measure of arterial stiffness and has shown to be a strong predictor of future cardiovascular events and mortality over traditional risk factors [2]. Cardiovascular magnetic resonance (CMR) allows for the evaluation of global stiffness and regional stiffness from distinct user-defined anatomical regions. Several methodologies have recently been validated for the non-invasive assessment of arterial stiffness using CMR, including total arterial compliance (TAC), a measure of global arterial stiffness [3,4], and ascending aortic distensibility (AD) [5,6] and aortic arch pulse wave velocity (PWV), measures of aortic stiffness [7-9]. However, the prognostic value of these CMR measures in the general population remains uncertain.

Arterial stiffness is a surrogate measure of end-organ disease and may represent an index of the summed effects of aging and risk factor exposure [1,2]. Different measures of arterial stiffness may have different prognostic value corresponding to differences in structural and geometric properties of the arterial wall [10]. For example, the impact of mechanical degeneration may have greatest effect in the more compliant, elastin-rich proximal aorta [11] while endothelial injury may largely impact stiffness in smaller muscular arteries [12]. Risk factors such as diabetes and hypercholesterolemia have been associated with increases in both central and peripheral stiffness [13], but associations between arterial stiffness and cardiovascular outcomes have differed depending on the site of arterial stiffness [14]. This has led to uncertainty over the most appropriate measure of arterial stiffness to apply in clinical practice and research trials.

The purpose of this study was therefore to evaluate comparative cardiovascular outcome associations among three CMR-derived measures of arterial stiffness within a large, multiethnic population free of clinical cardiovascular disease. In addition, we sought to evaluate associations between these CMR measures and traditional cardiovascular risk factors. We hypothesized that all three CMR measures of arterial stiffness would be predictive of future cardiovascular events, with possible differential associations with components of the outcome.

## Methods

### Study design and study participants

The Dallas Heart Study is a longitudinal, multiethnic, population-based probability sample of Dallas County residents. Details of the study design were previously described [15]. Briefly, from an initial cohort of 6101 subjects ages 18–65 who participated in a detailed survey regarding medical and family history, 3399 subjects ages 30–65 returned for a clinic visit involving blood and urine samples, and 2971 subsequently participated in a third visit for various imaging studies. Participants with baseline cardiovascular disease (defined as prior stroke, myocardial infarction, percutaneous coronary intervention, or coronary artery bypass grafting) were excluded (n = 638). The current study consisted of 2122 Dallas Heart Study participants who successfully underwent cardiovascular magnetic resonance (CMR). Written informed consent was obtained from all subjects, and the study was approved by the local Institutional Review Board.

### CMR protocol

**Figure 1:**
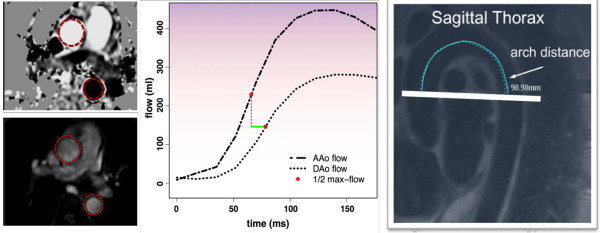
**CMR measures of aortic stiffness.***Left*, axial phase contrast images through the ascending and descending thoracic aorta. *Middle*, time-velocity flow curves through the ascending (AAo) and descending (DAo) thoracic aorta with measurement of transit time (*green line*) as the difference in time to half-max flow. *Right*, oblique sagittal view of the aortic arch with centerline measurement of aortic arch distance.

Participants underwent CMR using a 1.5 Tesla whole-body system (Intera, Philips Medical Systems, Best, Netherlands). All CMR studies were acquired with a four-element surface array coil. Ascending aortic distensibility (AD) and aortic arch pulse wave velocity (PWV) were assessed using a breath-hold, velocity-encoded, phase-contrast gradient echo sequence acquired perpendicular to the course of the ascending aorta 4 cm above the aortic valve plane (Figure 1) [16]. The ascending and descending thoracic aorta were imaged in cross-section with a temporal resolution of < 40 ms, 256 × 256 matrix, 34-cm field of view, 20° flip angle, 10 ms repetition time, 5 ms echo time, through-plane velocity encoding of ±150 cm/sec, and slice thickness 8 mm. Images were acquired using prospective ECG gating. The time-velocity curve was interpolated to a temporal resolution of 10 ms by using a cubic spine for subsequent analysis. The aortic arch was also evaluated with an oblique sagittal, double inversion-recovery spin echo image (“candy cane” view) with the following parameters: 33 cm field of view, electrocardiographically gated repetition time, 5.3 ms echo time, 32 echo train length.

Cardiac magnetic resonance, including a cine steady state free precession series of 10–13 short axis slices spanning the cardiac apex through the ventricular base, was acquired for measurement of left ventricular stroke volume (LVSV). Images were acquired during 15–20 second end-expiratory breath-holds using retrospective ECG gating. Additional parameters included: temporal resolution < 40 ms, 256 × 224 matrix (in-plane resolution 1.4 mm × 1.3 mm), 36-cm field of view, 70° flip angle, 4.2 ms repetition time, 2.1 ms echo time, 6 mm slice thickness and 4 mm slice gap.

### Blood pressure measurements

Brachial blood pressure was measured non-invasively using a non-ferromagnetic arm blood pressure cuff and automated blood pressure monitor. Four separate blood pressure measurements were acquired at various time points: (1) before scanning, outside the magnet, (2) before scanning, inside the magnet, (3) after scanning, inside the magnet, and (4) immediately after scanning, outside the magnet. The second and third blood pressure measurements were averaged for each subject. Average pulse pressure (PP) was calculated by the difference in average systolic blood pressure (SBP) and average diastolic blood pressure (DBP). In the case of participants whose blood pressures were not successfully measured during CMR, blood pressure measurements obtained closest in proximity to the time of CMR scanning were used for the brachial pulse pressure calculation.

### Calculation of arterial stiffness measures

Area contours of the ascending and descending thoracic aorta were manually traced through all phases of the cardiac cycle. Time-velocity flow curves of the ascending and descending thoracic aorta were produced using Qflow (v.4.1.6, Medis, Leesburg). Maximum and minimum cross-sectional areas of the ascending aorta were measured (Ao_max_ and Ao_min_, respectively). Transit time (t) through the aortic arch was calculated as the time between the ascending and descending upstroke velocities at half maximum [7]. A scout line indicating the position of the phase-contrast acquisition image plane was displayed on the candy cane view of the aortic arch, and arch distance (d) was determined by drawing a freehand line through the center of the aorta parallel to the aortic walls between the positions in the ascending and descending aorta at which arterial flow was measured, as indicated by the scout line.

Total arterial compliance (TAC) [4] was calculated by dividing left ventricular stroke volume (LVSV) by average pulse pressure (PP):

$$\mathrm{TAC}=\frac{\Delta \;\mathrm{Volume}}{\mathrm{\Delta Pressure}}=\frac{\mathrm{LVSV}}{\mathrm{PP}}$$

Ascending aortic distensibility (AD) [17] was calculated using the following equation:

$$\mathrm{AD}=\frac{\left[Ao_{\max}-Ao_{\min}\right]}{\mathrm{PP}\times Ao_{\min}}$$

Arch pulse wave velocity (PWV) [16,18] was calculated by dividing arch distance (d) by transit time (t):

$$\mathrm{PWV}=\frac{d}{t}$$

### Coronary artery calcium scores

Coronary artery calcium (CAC) scores were measured using an Imatron C-150XP electron-beam CT scanner, with results expressed in Agatston units [19]. Duplicate scans were obtained 1–2 minutes apart, and the results were averaged. A score >10 Agatston units was selected as a data-derived threshold to define the presence of coronary artery calcium in order to maximize signal-to-noise ratio and reproducibility.

### Cardiovascular events & surveillance

Subjects were followed for incident cardiovascular events over a mean period of 7.8 ± 1.5 years. Fatal cardiovascular events were ascertained using the National Death Index. Deaths were classified as cardiovascular if they included International Statistical Classification of Diseases, 10th Revision codes I00-I99 [20]. Subjects were contacted annually to participate in a detailed health survey in regard to interval nonfatal cardiovascular events. In addition, all subjects were tracked quarterly for hospital admissions by using the Dallas-Fort Worth Hospital Council Data Initiative database, which includes hospital claims for 77 hospitals across 28 counties in North Texas and represents 90% of the health care market volume in this region [21]. Nonfatal cardiovascular events assessed included: 1) nonfatal cardiac events (nonfatal myocardial infarction, hospitalization for unstable angina, coronary revascularization including percutaneous revascularization and coronary artery bypass graft surgery, hospitalization due to congestive heart failure, and hospitalization for atrial fibrillation); and 2) nonfatal extra-cardiac vascular events. Nonfatal extra-cardiac vascular events include both nonfatal cerebrovascular events (nonfatal stroke, transient ischemic attack, cerebrovascular revascularization) and nonfatal peripheral vascular events (peripheral arterial revascularization and abdominal aortic aneurysm repair). Primary records were requested for all suspected cardiovascular events and these events were adjudicated separately by two cardiologists [22]. Subjects lost to follow-up (n = 210) were excluded from the analysis cohort.

### Covariate definitions

Hypertension was defined as systolic blood pressure ≥140 mm Hg, diastolic blood pressure ≥ 90 mm Hg, or use of baseline blood pressure lowering medication. Hypercholesterolemia was defined as fasting low density lipoprotein cholesterol ≥100 mg/dL or fasting total cholesterol ≥200 mg/dL. Diabetes mellitus was defined as a fasting glucose ≥125 mg/dL or use of hypoglycemic medications. Body mass index (BMI) was calculated using the equation weight (kg)/height (m^2^). Subject gender, ethnicity and cigarette smoking were determined by self report. Mean abdominal aortic wall thickness (MAWT) was measured by CMR as previously described [22].

### Statistics

TAC, AD, and PWV were analyzed as categorical variables (gender and ethnicity-specific quartiles) and as continuous variables. Multivariate linear regression was used to evaluate associations between traditional risk factors and CMR measures of arterial stiffness. Kaplan-Meyer cumulative incidence curves were constructed for incident cardiovascular events. The primary endpoint was composite events (combined nonfatal cardiac events, nonfatal extra-cardiac vascular events, and cardiovascular death). Secondary endpoints were 1) cardiovascular death, 2) nonfatal cardiac events, and 3) nonfatal extra-cardiac vascular events. Cox proportional hazards regression was used to assess associations of TAC, AD, and PWV in relation to incident events after adjustment for age, sex, ethnicity, body mass index, systolic blood pressure, use of blood pressure lowering medications, resting heart rate, diabetes mellitus, cigarette smoking, and hypercholesterolemia. The Supremum test confirmed a linear term for age was most appropriate in our adjusted Cox proportional hazard models. The contribution of CMR-derived arterial stiffness measures to cardiovascular risk models including the Framingham Risk Score were assessed using the likelihood ratio chi-square statistic [23]. Raw percentages of primary and secondary endpoints were assessed by the Cochran-Armitage trend test. A value of p < 0.05 was considered statistically significant. All statistical analyses were performed with SAS (version 9.0, SAS Institute Inc., Cary, NC).

## Results

Baseline characteristics of the study population are presented (Table 1). During the surveillance period, 153 subjects (6.9%) experienced a first cardiovascular event, of which 38 subjects (1.6%) succumbed to cardiovascular death, 81 subjects (3.7%) experienced a nonfatal cardiac event, and 47 subjects (2.2%) experienced a nonfatal extra-cardiac vascular event.

**Table 1:** Baseline characteristics of the study population

	**(n** = **2122)**
Age (years)	44 ± 10
Body mass index (kg/m2)	30 ± 7
Systolic blood pressure (mmHg)	126 ± 17
Use of blood pressure lowering medication (%)	19%
Resting heart rate (beats per minute)	75 ± 11
Hypertension (%)	29%
Male sex (%)	44%
Ethnicity	
African American	49%
White	33%
Hispanic	16%
Other	2%
Diabetes (%)	10%
Current smoking (%)	26%
Hypercholesterolemia (%)	12%
Low HDL-C (%)	39%
Family history of myocardial infarction (%)	31%
Coronary artery calcium score >10 (%)	19%
Mean abdominal aortic wall thickness (mm)	1.68 ± 0.30
Total arterial compliance (ml/mmHg)	1.60 ± 0.46
Ascending aortic distensibility (mmHg^−1^ × 10^−3^)	5.0 ± 3.1
Aortic arch pulse wave velocity (m/s)	4.9 ± 3.0

No differences in CMR measures of arterial stiffness were observed between subjects in the analysis cohort (n = 2122) and subjects lost to follow-up (n = 211), all p > 0.20. Increasing age, systolic blood pressure, and resting heart rate were independently associated with lower TAC, lower AD, and faster PWV (all p < .0001) (Table 2). Male gender was independently associated with lower AD (p < .0001), but not with either PWV (p = 0.25) or TAC (p = 0.89). Diabetes was independently associated with lower TAC (p = 0.0002) and lower AD (p = 0.04), but not with PWV (p = 0.60). Increasing body mass index was independently associated with lower TAC (p < .0001) and faster PWV (p = 0.0004), but not with AD (p = 0.16).

**Table 2:** Linear regression associations between traditional risk factors and CMR measures of arterial stiffness

**Variable**	**Ascending aortic distensibility ΔmmHg × 10**^**−3**^				**Arch pulse wave velocity Δlog(m/s)**				**Total arterial compliance Δml/mmHg**			
	**Univariate**		Multivariate*		**Univariate**		Multivariate*		**Univariate**		Multivariate*	
	**β**	**P**	**β**	**P**	**β**	**P**	**β**	**P**	**β**	**P**	**β**	**P**
Age (per 10 year increase)	−1.9	<.0001	−1.5	<.0001	0.25	<.0001	0.20	<.0001	−0.20	<.0001	−0.13	<.0001
Gender (male)	−1.0	<.0001	−0.83	<.0001	0.04	0.01	0.02	0.25	0.12	<.0001	0.003	0.89
Ethnicity (African American)	−0.82	<.0001	−0.04	0.68	0.16	<.0001	0.06	<.0001	−0.14			
SBP (per 10 mmHg increase)	−0.98	<.0001	−0.56	<.0001	0.11	<.0001	0.07	<.0001	−0.14	<.0001	−0.11	<.0001
Use of hypertension medications	−2.3	<.0001	−0.25	0.20	0.28	<.0001	0.03	0.13	−0.31	<.0001	−0.01	0.72
Resting heart rate (per 10 beat per minute increase)	−0.39	<.0001	−0.38	<.0001	0.018	0.01	0.027	<.0001	−0.06	<.0001	−0.05	<.0001
Current smoking	−0.19	0.17	0.002	0.98	0.06	0.0008	0.02	0.37	0.005	0.87	−0.001	0.94
Diabetes mellitus	−2.2	<.0001	−0.32	0.04	0.18	<.0001	−0.01	0.60	−0.41	<.0001	−0.11	0.0002
Total cholesterol (per 10 mg/dL increase)	−0.12	<.0001	−0.11	0.28	0.014	<.0001	0.001	0.66	−0.014	<.0001	−0.005	0.03
HDL-C (per 10 mg/dL increase)	0.08	0.04	−0.10	0.76	0.02	0.0005	0.016	0.003	0.006	0.50	0.016	0.009
BMI (per 1 kg/m^2^ increase)	−0.06	<.0001	−0.01	0.16	0.0005 =	0.96	−0.005	0.0004	−0.00	0.12	0.013	<.0001

β=beta coefficient for linear regression.

P=statistical significance.

*Multivariate analyses are adjusted for all covariates listed in the first column.

**Figure 2:**
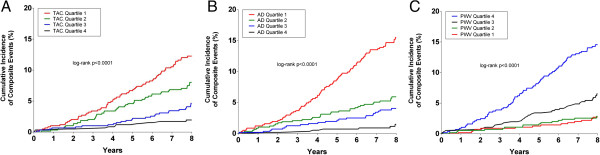
**Cumulative incidence curves for composite cardiovascular events based on quartile of (A) total arterial compliance, (B) ascending aortic distensibility, and (C) arch pulse wave velocity.**

Progression from highest to lowest quartiles of TAC and AD, and from lowest to highest quartile of PWV were associated with incremental increased risk for composite cardiovascular events (Figure 2). When analyzed as continuous variables, changes in TAC, AD, and PWV were each associated with increased risk for composite events (all p < .0001). After adjusting for traditional risk factors, TAC maintained a modest association with composite events (HR 1.07 per 1SD change, 95% CI 1.01-1.14, p = 0.03) (Table 3), while the association between AD and composite events trended towards significance (HR 1.18, 95% CI 0.95-1.46, p = 0.08). PWV was not independently associated with composite events after adjusting for traditional risk factors (HR 1.11 per 1SD change, 95% CI 0.89-1.32, p = 0.28). However, PWV independently conferred increased risk for nonfatal extra-cardiac vascular events (HR 1.18 per 1SD change, 95% CI 1.02-1.55, p = 0.04). Neither TAC nor AD were independently associated with nonfatal extra-cardiac vascular events after multivariable adjustment. However, TAC and AD independently conferred increased risk for nonfatal cardiac events (HR 1.11 per 1SD change, 95% CI 1.04-1.19, p = 0.001; and HR 1.45 per 1SD change, 95% CI 1.18-1.78, p = 0.0005, respectively).

**Table 3:** Hazard ratios for cardiovascular events

	**Unadjusted**			Adjusted*		
**Total arterial compliance**^**†**^	**HR**	**95% CI**	**P**	**HR**	**95% CI**	**P**
Composite events	1.14	1.10-1.18	<.0001	1.07	1.01-1.14	0.03
Cardiovascular death	1.10	1.01-1.20	0.02	0.97	0.73-1.30	0.85
Nonfatal cardiac events	1.15	1.10-1.20	<.0001	1.11	1.04-1.19	0.001
Nonfatal extra-cardiac vascular events	1.10	1.02-1.19	0.02	0.97	0.74-1.28	0.86
**Ascending aortic distensibility**^**‡**^						
Composite events	1.63	1.50-1.77	<.0001	1.18	0.95-1.46	0.08
Cardiovascular death	1.49	1.25-1.77	<.0001	0.72	0.41-1.28	0.27
Nonfatal cardiac events	1.69	1.52-1.87	<.0001	1.45	1.18-1.78	0.0005
Nonfatal extra-cardiac vascular events	1.52	1.31-1.76	<.0001	1.09	0.76-1.57	0.63
**Arch pulse wave velocity**^**§**^						
Composite events	1.23	1.15-1.31	<.0001	1.11	0.89-1.32	0.28
Cardiovascular death	1.25	1.11-1.41	<.0001	0.92	0.63-1.33	0.64
Nonfatal cardiac events	1.19	1.08-1.32	<.0001	1.00	0.76-1.32	0.97
Nonfatal extra-cardiac vascular events	1.76	1.24-2.11	<.0001	1.18	1.02-1.55	0.04

*adjusted for age, sex, ethnicity, systolic blood pressure, use of blood pressure medication, resting heart rate, diabetes mellitus, current smoking, body mass index, and hypercholesterolemia.

^†^hazard ratios per 1SD (0.46 ml/mmHg) decrease in total arterial compliance.

^‡^hazard ratios per 1SD (3.1 mmHg^−1^ × 10^−3^) decrease in aortic distensibility.

^§^hazard ratios per 1SD (3.0 m/s) increase in pulse wave velocity.

When PWV was included in a model with the Framingham Risk Score, there was a modest increase in the C statistic for composite events which approached significance (0.771 vs. 0.755, p = 0.05). Although modest increases in the C statistic were also observed when AD and TAC were included in models with the Framingham Risk Score, these differences were not significant (0.772 vs. 0.755, p = 0.55 and 0.769 vs. 0.755, p = 0.36, respectively).

## Discussion

Our study is the first to evaluate the prognostic value of CMR-derived measures of TAC, AD, and PWV within a large, multiethnic population without clinical cardiovascular disease. Results from our study demonstrate that all three measures are associated with future cardiovascular events. After adjusting for traditional risk factors, TAC remained independently predictive of composite events, while the relationship between AD and composite events approached significance. Although PWV was not independently predictive of composite events, changes in PWV were independently associated with nonfatal extra-cardiac vascular events. Further, incorporation of PWV into a risk model with the Framingham Risk Score yielded modest improvement in the C statistic which approached significance (p = 0.05).

Arterial stiffness, a measure of vascular function, is central to the onset of atherosclerosis and is a primary determinant of age-related increases in systolic blood pressure and pulse pressure [24]. Aortic stiffening is a major contributor to arterial stiffness, and several prior population studies have documented a strong independent predictive role of aortic stiffness, measured by carotid-femoral PWV, with respect to cardiovascular morbidity and mortality. For example, in the Rotterdam Study, Mattace-Raso et al [25] used pressure transducer-derived carotid-femoral PWV to demonstrate that the hazard ratio for coronary heart disease among subjects in the highest tertile of PWV was 2.45 after adjusting for age, gender, mean arterial pressure, and heart rate. Likewise, in the Framingham Heart Study, Mitchell et al [26] demonstrated that faster carotid-femoral PWV conferred increased risk for an initial cardiovascular event (HR 1.48 per SD increase) after adjusting for traditional risk factors. In the Health ABC Study, Sutton-Tyrrell et al [27] demonstrated that Doppler flow-derived carotid-femoral PWV was independently associated with greater cardiovascular mortality and stroke among a large cohort of healthy adults. Carotid-femoral PWV has further been shown to improve risk stratification among patients already identified as having higher baseline cardiovascular risk, such as those with end-stage renal disease [28], and hypertension [29].

However, the prognostic value of CMR measures of arterial stiffness has not been explored in prior studies.

There are several advantages of CMR for the noninvasive assessment of arterial stiffness. First, CMR has capability to measure local and regional arterial stiffness at multiple vascular segments during a single CMR imaging session. In addition, CMR affords freedom from operator bias and offers accurate determination of path length for the calculation of PWV, which is not available using other modalities [30]. The current technique for measuring aortic stiffness using tonometry requires specialized equipment and training which limits its clinical utility. CMR-derived TAC [31,32], ascending AD [3,33], and arch PWV [3,18] have each been validated against traditional methods for assessing arterial stiffness and demonstrate high reproducibility. Further, all three measures are easily obtained during routine CMR without the need for specialized post-processing software. CMR-derived measures are readily standardized to facilitate comparability to other modalities and evaluate changes over time.

While assessment of arterial stiffness is suggested to improve risk stratification as a measure of end-organ disease [34], it is important to note that susceptibility to insult from cardiovascular risk factors differs among different arterial segments [4]. Since arterial stiffness may be a marker of cumulative insult, having different susceptibilities to insults at specific arterial segments may have important implications for predicting cardiovascular outcomes. Our results highlight similarities and differences for risk factor associations among CMR measures of arterial stiffness. Age, systolic blood pressure, and heart rate were determinants of all three CMR measures, which is concordant with prior studies [17,35,36]. However, differences in risk factor susceptibility were also observed among our CMR measures. For example, we observed African American ethnicity to be an independent determinant of lower TAC and faster PWV, but not lower AD. This finding is discordant from prior observations by Malayeri et al [35] who reported a significant association between African American ethnicity and lower AD within the MESA cohort. This discrepancy may be accounted for by differences in our multivariate models, as our study included resting heart rate and diabetes mellitus as covariates, both of which are independent determinants of AD. Further, we observed diabetes mellitus was independently associated with lower TAC and lower AD, but not with faster PWV. Stacey et al [37] described a similar association between diabetes and AD within the MESA cohort. While the pathohysiologic mechanism for this observation remains unclear, elevated glycemic status may have a preferential deleterious effect on the elastin-rich ascending aorta and a lesser effect on distal aortic segments.

While TAC was the only CMR measure which demonstrated a significant independent association with our primary endpoint of composite events, the relationship between AD and composite events approached significance (p = 0.08). However, our results also suggest that different measures of arterial stiffness by CMR may be associated with different subtypes of cardiovascular outcomes. For example, both TAC and AD conferred independent increased risk for nonfatal cardiac events but not nonfatal extra-cardiac vascular events. In contrast, PWV conferred independent increased risk for nonfatal extra-cardiac vascular events but not nonfatal cardiac events. None of our CMR measures were predictive of cardiovascular death after adjusting for traditional risk factors. These results suggest CMR measures of local and regional aortic stiffness (AD and PWV, respectively) and global arterial stiffness (measured by TAC) may have distinct sensitivities for evaluating the downstream pathologic manifestations of arterial stiffening. For example, processes resulting in lower TAC and lower AD may preferentially manifest through deleterious effects on the heart, whereas process resulting in faster PWV may preferentially manifest through deleterious effects on the brain and distal arteries. Prior work from our lab demonstrated PWV is a strong independent predictor of chronic ischemic insult to the brain, as indicated by white matter hyperintensity volume [16]. Thus, different measures of arterial stiffness may function as better surrogate markers of end-organ disease in different organ systems.

There was modest increase in the C statistic by including PWV in a model with the Framingham Risk Score which approached significance. However, no significant increment in the C statistic was observed by including TAC or AD in similar models. Thus, our results do not encourage widespread screening for cardiovascular risk using solely CMR stiffness in the general population. However, CMR measures of arterial stiffness can be easily obtained during routine CMR exams and can potentially serve as useful subclinical measures to investigate different phases of cardiovascular disease and end-organ damage. Future studies are necessary to compare the incremental prognostic value of CMR measures of arterial stiffness with markers of atherosclerosis burden, such as coronary artery calcium score.

### Limitations

There were several limitations of this study. First, a noninvasive measure of peripheral blood pressure was used to calculate AD and TAC instead of an invasive measure of central aortic pressure. Despite this limitation, prior studies have indicated that noninvasive blood pressure measures are adequate approximations and predict future cardiovascular events [38,39]. Second, a limited number of adverse events throughout the surveillance period limited our statistical power to explore associations with event subtypes and within subgroups. Thus, the present data may underestimate the predictive value of arterial stiffness, and future studies with longer surveillance periods are necessary to examine the prognostic value of these measures among patients with different baseline cardiovascular risk. Since the number of observed nonfatal extra-cardiac vascular events was particularly low during the surveillance period (n = 47), nonfatal cerebrovascular events were combined with nonfatal peripheral vascular events into a single event subcategory so as to improve power for multivariate regression analyses. However, arterial stiffening at different vascular sites may elicit differential influences on one’s risk for future cerebrovascular and peripheral vascular events. Future studies are necessary to examine associations between CMR measures of arterial stiffness and nonfatal cerebrovascular and peripheral vascular events as separate outcome categories. Third, since our study population was free of clinical cardiovascular disease, the observed prognostic value of CMR-derived stiffness measures may be lower. A recent meta-analysis by Vlachopoulos et al [2] demonstrated the predictive ability of carotid-femoral PWV was greatest among subjects with higher baseline cardiovascular risk. Future studies are necessary to examine the prognostic value of CMR measures among higher risk subgroups. Forth, TAC is a crude indicator of global arterial stiffness based on a modified two-factor Windkessel model combining resistance and capacitance. As such, TAC is limited in describing all components of arterial mechanics [40]. Finally, there were limitations of our 2D phase contrast sequence used to calculate PWV and AD. For example, no through-plane motion correction was used during the acquisition, which likely compromised area measurements of the ascending aorta [41]. Adoption of a more advanced phase contrast technique with higher temporal resolution and through-plane motion correction would circumvent this limitation in future studies [42].

## Conclusions

In conclusion, we demonstrated that CMR measures of TAC, AD, and PWV are associated with adverse cardiovascular events. Further, different CMR measures appear to have different associations with traditional risk factors and specific subtypes of cardiovascular outcomes. While TAC and AD demonstrated stronger associations with nonfatal cardiac events, PWV demonstrated a stronger association with nonfatal extra-cardiac vascular events. Future studies are necessary to evaluate the prognostic value of these measures for earlier subclinical markers of end-organ disease. In addition, the prognostic value of these CMR measures should be explored among subjects with higher baseline cardiovascular risk.

## Abbreviations

AAo: Ascending thoracic aorta; AD: Ascending aortic distensibility; BMI: Body mass index; CMR: Cardiovascular magnetic resonance; DAo: Descending thoracic aorta; DBP: Diastolic blood pressure; ECG: Electrocardiogram; HR: Hazard ratio; LVSV: Left ventricular stroke volume; MAWT: Mean abdominal aortic wall thickness; CMR: Cardiovascular Magnetic resonance; PP: Pulse pressure; PWV: Arch pulse wave velocity; SBP: Systolic blood pressure; SD: Standard deviation; TAC: Total arterial compliance.

## Competing interests

The authors declare that they have no competing interests.

## Authors’ contributions

CM, AK, and KK contributed with writing and editing of the manuscript, experimental design, and CMR CA contributed with statistical design and analysis, and with editing of the manuscript AG and SA contributed with data analysis and editing of the manuscript RP contributed with editing of the manuscript, experimental design, and CMR. All authors read and approved the final manuscript.
